# Protein Helps Orchestrate Cells' Fluid Uptake

**DOI:** 10.1371/journal.pbio.0020318

**Published:** 2004-08-24

You are what you eat and drink. Steak can sit in your stomach or orange juice wind through your intestines, but they only become part of your body once they're taken up by your cells. First, foods must be reduced to a soup of proteins, fats, sugars, and so on. But even then, getting these materials into a cell isn't as simple as sticking them in your mouth. For one, there's the membrane enclosing a cell. Simply puncturing a hole in the membrane would spill the cell's contents, harming or killing the cell.

Instead, all eukaryotes—organisms whose cells have nuclei—use a carefully orchestrated process called endocytosis to bring materials into their cells. Eukaryotic cells first form cavities in their cell membrane that surround nearby particles or fluid. These pockets seal shut and bud off into the cell to form small membrane-bound sacs called vesicles.

When taking in fluids, eukaryotic cells use two distinct mechanisms—to take tiny sips or huge gulps. With one process, called pinocytosis, cells continually form small pockets in the cell membrane that enclose small droplets of fluid in vesicles called pinosomes. These newly formed vesicles, called early endosomes, bud off from the membrane and fuse with other early endosomes. In one form of pinocytosis, the vesicles are encaged by a protein called clathrin that tightly constrains their size. These carriers incorporate membrane constituents (for example, growth factors) with very high selectivity. In macropinocytosis, on the other hand, large ruffles in the membrane engulf mass quantities of fluid in vesicles known as macropinosomes.

Beyond taking in nutrients, these processes are essential to the function of many organs—from the brain, where nerve cells receive other cells' chemical signals by pinocytosis, to the kidney, where cells use macropinocytosis to take in waste fluids for processing. Macropinocytosis is also relevant to cancer cells; it has long been known that oncogenes dramatically induce this endocytic process, affecting the signaling status of these cells. But compared with other types of endocytosis, molecular biologists know surprisingly little of the mechanisms behind macropinocytosis. They do know that the Rab5 protein—an enzyme that coordinates a complex network of other proteins, called effectors—is crucial for both pinocytosis and macropinocytosis.

Now, as reported in this issue of *PLoS Biology*, Marino Zerial and colleagues have found a new protein, which they named Rabankyrin-5, that forms a further link between these two mechanisms for fluid uptake. The protein is necessary for macropinocytosis, and its levels control the rate of this process. In addition, Rabankyrin-5 helps regulate endosome trafficking and coordinates this mechanism with macropinocytosis.

In two commonly used human and mouse cell lines, the researchers found the protein Rabankyrin-5 along with Rab5 on both types of pinosomes, early endosomes and macropinosomes. The early endosomes usually fuse with one another inside the cell, but when the researchers blocked Rabankyrin-5 activity, this fusion fell sharply. Suppressing Rabankyrin-5 activity also stifled macropinocytosis; overexpressing the effector, on the other hand, sent macropinocytosis into overdrive.

The researchers also looked at endocytosis in mouse kidney and canine kidney cell lines. Inside the kidney, fluid-carrying ducts are lined with epithelial cells that take up liquids through their exposed surface. The researchers found Rabankyrin-5 predominately on vesicles at this surface, and as in the other experiments, overexpression of the protein promoted macropinocytosis. Together, these findings suggest Rabankyrin-5 plays a role in regulating this form of fluid uptake and plays a role in kidney function. The discovery of Rabankyrin-5 involvement in macropinocytosis also has implications for other physiological and pathological mechanisms such as the immune system response, defense against pathogens, and hyperactivation of signaling pathways in cancer cells.

Rabankyrin-5 contains various regions that bind other proteins and also lipids found in cell membranes, suggesting the protein plays a mechanical role in forming vesicles. The protein also has regions found on other proteins that are involved in signaling and development, so it may help direct vesicles' traffic within the cell. The protein also has regions characteristic of proteins involved in clathrin-dependent endocytosis, which fits with the researchers' finding that Rabankyrin-5 affects pinocytosis.[Fig pbio-0020318-g001]

**Figure pbio-0020318-g001:**
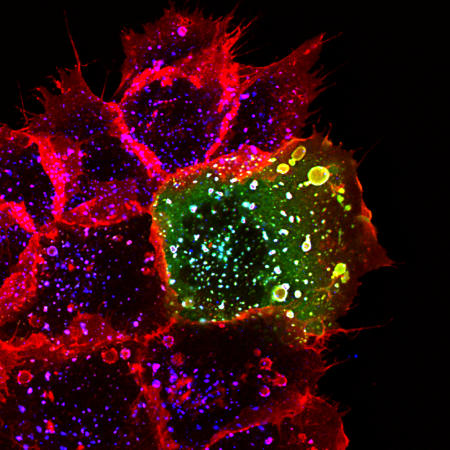
Rabankyrin-5 (green) colocalizes with rhodamine-conjugated EGF on macropinosomes after growth factor stimulation

All told, Rabankyrin-5 appears to form a bridge between two distinct mechanisms, pinocytosis and macropinocytosis, that cells use to take in fluids. While the details of how Rabankyrin-5 functions are still unclear, these findings give researchers a new handle for grasping how macropinocytosis works and how cells control when and how much they drink in their surroundings.

